# On-Site Stimulation of Dendritic Cells by Cancer-Derived Extracellular Vesicles on a Core–Shell Nanowire Platform

**DOI:** 10.1021/acsami.4c00283

**Published:** 2024-05-28

**Authors:** Min Zhang, Miki Ono, Shota Kawaguchi, Mikiko Iida, Kunanon Chattrairat, Zetao Zhu, Kazuki Nagashima, Takeshi Yanagida, Junya Yamaguchi, Hiroyoshi Nishikawa, Atsushi Natsume, Yoshinobu Baba, Takao Yasui

**Affiliations:** † Department of Biomolecular Engineering, Graduate School of Engineering, Nagoya University, Furo-cho, Chikusa-ku, Nagoya 464-8603, Japan; ‡ Department of Life Science and Technology, Tokyo Institute of Technology, Nagatsuta 4259, Midori-ku, Yokohama 226-8501, Japan; § Research Institute for Electronic Science (RIES), Hokkaido University, Kita, Sapporo, Hokkaido 001-0020, Japan; ∥ Department of Applied Chemistry, Graduate School of Engineering, The University of Tokyo, 7-3-1 Hongo, Bunkyo-ku, Tokyo 113-8656, Japan; ⊥ Department of Immunology, Nagoya University Graduate School of Medicine, Nagoya 466-8550, Japan; # Division of Cancer Immunology, Exploratory Oncology Research and Clinical Trial Center (EPOC), National Cancer Center, Chiba 277-8577, Japan; ¶ Institute of Nano-Life-Systems, Institutes of Innovation for Future Society, Nagoya University, Furo-cho, Chikusa-ku, Nagoya 464-8603, Japan; ∇ Kawamura Medical Society, Gifu 501-3144, Japan; ○ Institute for Quantum Life Science, National Institutes for Quantum Science and Technology (QST), Anagawa 4-9-1, Inage-ku, Chiba 263-8555, Japan

**Keywords:** ZnO/Al_2_O_3_ core−shell nanowire platform, extracellular vesicles, dendritic cells, antigen presentation, nanowires, immunotherapy

## Abstract

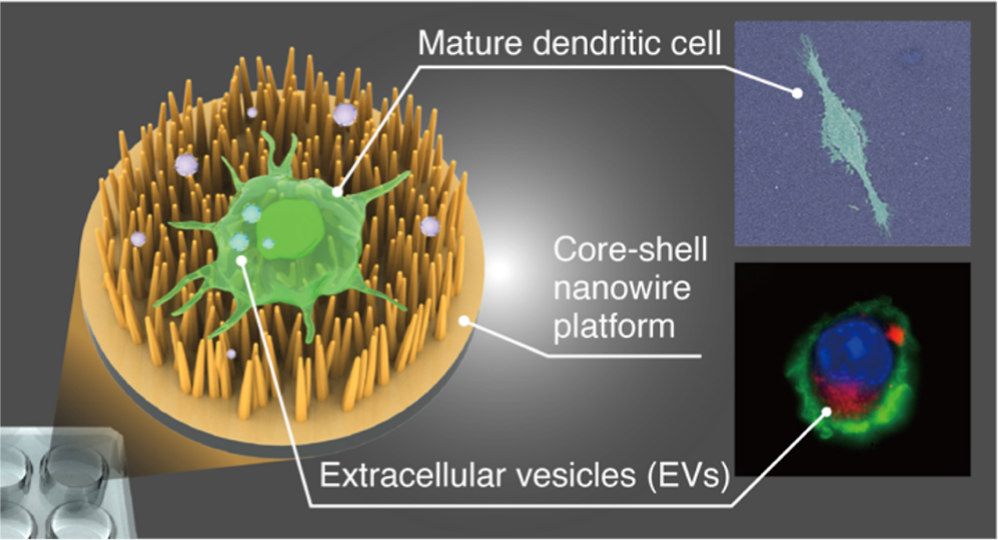

Extracellular vesicles (EVs) contain a subset of proteins, lipids, and nucleic acids that maintain the characteristics of the parent cell. Immunotherapy using EVs has become a focus of research due to their unique features and bioinspired applications in cancer treatment. Unlike conventional immunotherapy using tumor fragments, EVs can be easily obtained from bodily fluids without invasive actions. We previously fabricated nanowire devices that were specialized for EV collection, but they were not suitable for cell culturing. In this study, we fabricated a ZnO/Al_2_O_3_ core–shell nanowire platform that could collect more than 60% of the EVs from the cell supernatant. Additionally, we could continue to culture dendritic cells (DCs) on the platform as an artificial lymph node to investigate cell maturation into antigen-presenting cells. Finally, using this platform, we reproduced a series of on-site immune processes that are among the pivotal immune functions of DCs and include such processes as antigen uptake, antigen presentation, and endocytosis of cancer-derived EVs. This platform provides a new ex vivo tool for EV-DC-mediated immunotherapies.

## Introduction

Cancer immunotherapy is becoming one of the most promising new cancer treatment approaches due to its advantages of being noninvasive and offering simultaneous treatments of multiple sites at a low cost, compared to conventional cancer treatments such as surgery, radiation therapy, and chemotherapy, which are highly invasive, single-site treatments that come at a high cost. Cancer immunotherapy involves enhancing the ability of a patient’s own immune system by activating it, and dendritic cells (DCs) play a crucial role as powerful antigen-presenting cells (APCs) in immunotherapy. DCs take up antigens, mainly in the form of peptides with cancer antigens or RNA or DNA taken from tumors.^1,2^ However, surgery is required to obtain the tumors, and a problem of type incompatibility arises if the tumors are not from the patient’s own tumors.

To achieve nonsurgical cancer immunotherapy, a promising approach is the use of extracellular vesicles (EVs) for both early cancer diagnosis and immunotherapy. EVs, which are small membrane vesicles ranging in size from 30 to 200 nm, play a critical role in cell-to-cell communication,^3−6^ and they can be easily and noninvasively isolated from body fluids. Cancer cells secrete more EVs carrying cancer-specific messenger molecules than their nonmalignant counterparts,^7,8^ and extensive research has been conducted on the use of EVs in cancer diagnosis and treatment.^9−11^ Studies have shown that differences between cell-free microRNAs (miRNAs)^12^ and miRNAs contained in EVs^13−15^ can be detected between nondisease and disease states at an early stage. Moreover, tumor cell-derived EVs have been shown to transmit tumor-specific major histocompatibility complex (MHC) molecules and contribute to antigen presentation, thereby promoting immune recognition.^16,17^ While antigen presentation using EVs has attracted some attention,^18−25^ previous reports of effective EV-based immunotherapy have used ascites-derived EVs,^16^ which require invasive procedures. Therefore, in this study, we propose using nanowires not only for EV capture but also for EV-based antigen presentation for immunotherapy in vitro, which may overcome the limitations of invasive procedures and facilitate the development of nonsurgical cancer immunotherapy.

Taking the viewpoint of the advantages of high efficiency in capturing EVs using nanowires,^26^ we have considered the potential application of nanowires in cancer immunotherapy in vitro*.* We have previously reported on the use of nanowires for early cancer diagnosis through the capture and analysis of EVs from urine,^27−29^ and a high efficiency in a charge-based manner has been obtained.^30,31^ Given the potential of this nanowire-based methodology for biomedical applications, it is essential to develop an EV-collecting nanointerface to design the next generation of therapeutic platforms. Although nanowires have shown great potential in analyzing cell properties, to our knowledge, there have been fewer studies on applications that culture DCs on nanowires. We have previously reported that the ZnO/Al_2_O_3_ core–shell nanowire can capture a high number of EVs from the biological samples by leveraging the electrostatic interaction between negatively charged biomolecules and positively charged nanowires^29,32^ Here, we have fabricated a ZnO/Al_2_O_3_ core–shell nanowire platform that enables both EV capture and subsequently cell culture. We have observed DC proliferation and differentiation on the nanowire platform and confirmed the phagocytosis of cancer-cell-derived EVs by DCs. The growth of DCs on nanowires and their ability to perform the cellular functions there as initiators in promoting an immune response against pathogenic or self-tumor antigens led us to recognize the potential for immunotherapy using nanowires.

## Results and Discussion

### EV Capture and Cytotoxicity Evaluation of the Nanowire Platform

**Figure 1:**
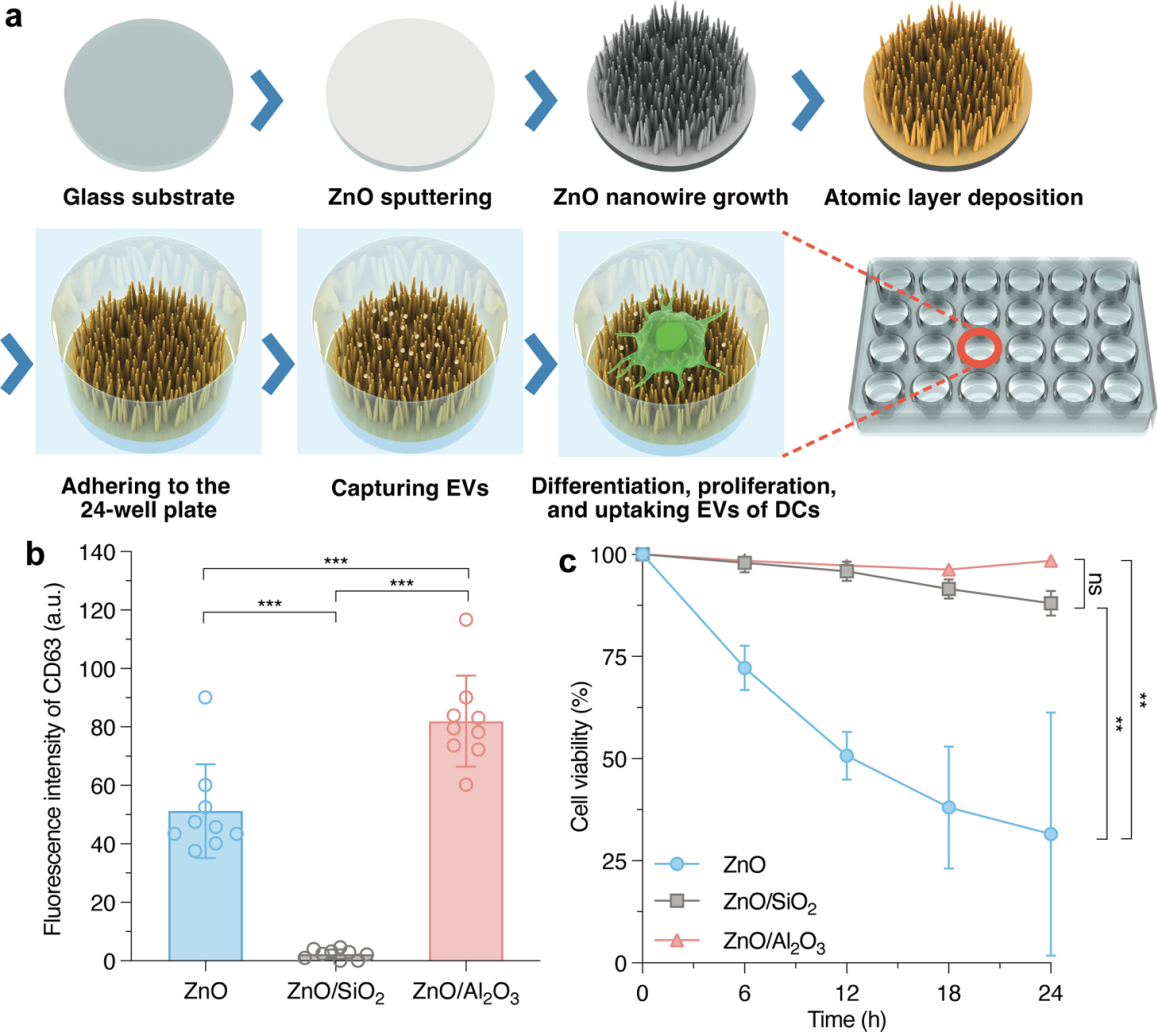
Fabrication and evaluation of the nanowire platform. (a) Schematic illustration of the nanowire platform fabrication method. (b) CD63 detection of EVs on nanowires with three different nanowire types. Error bars show the SD for an individual experiment (*N* = 9). (c) GL261 cell viability over time on nanowires with three different nanowire types. Error bars show the SD for an individual experiment (*N* = 3). In (b,c), the *p* value was calculated by an unpaired two-tailed *t*-test (**, *p* < 0.05; ***, *p* < 0.001, and ns, not significant).

The key point of the immunotherapy platform is to provide both high-efficiency capture of EVs and a stable environment suitable for culturing cells. We previously reported several oxide nanowire microfluidic devices which could capture EVs, RNA, or ssDNA with high efficiency,^27−31,33−37^ but they lacked cell culture capability. In this study, we used zinc oxide (ZnO) nanowires as the core template, and a thin film of different metal oxides as the shell was deposited on the ZnO nanowires using the atomic layer deposition (ALD) method to fabricate the platform for the differentiation and proliferation of immune cells. A glass substrate on which the ZnO nanowires were grown and got an oxide layer by ALD was adhered to the bottom of a 24-well plate to obtain the core–shell nanowire platform (Figure 1a), while only a glass substrate adhered to the bottom of a 24-well plate was the glass well platform. To investigate whether it would be possible to capture EVs and culture cells simultaneously, we measured CD63 which is a biomarker for EVs^38−40^ and cell viability.

The ZnO/Al_2_O_3_ core–shell nanowire platform had the strongest fluorescence for CD63, a small EV marker,^41^ which meant that the ZnO/Al_2_O_3_ core–shell nanowires captured the most EVs among the three types of nanowires, ZnO, ZnO/SiO_2_, and ZnO/Al_2_O_3_ (Figure 1b). The cell viability of the different nanowire platforms showed that the ZnO nanowire platform had a time-dependent decrease in cell viability, which is consistent with the literature,^42^ while the ZnO/SiO_2_ and the ZnO/Al_2_O_3_ nanowire platforms did not (Figure 1c). In addition, the ZnO/Al_2_O_3_ nanowire platform even showed cell proliferation at 24 h. Scanning transmission electron microscopy images and energy dispersive X-ray spectroscopy elemental mappings (Figure S1) confirmed that the core/shell structure was obtained for the ZnO/Al_2_O_3_ nanowires. According to the parameters shown in Figure S2a,b, 50 ALD cycles had a higher capture rate of EVs (Figure S2c). The size distribution and the observed number of EVs led us to believe that the ZnO/Al_2_O_3_ nanowires with 50 ALD cycles captured the 30–300 nm EVs (Figure S2d). As a result, ZnO/Al_2_O_3_ nanowires obtained with 50 ALD cycles were the optimal metal oxides that simultaneously satisfied the required capture of EVs and cell culture. We selected the ZnO/Al_2_O_3_ core–shell nanowire platform for further research.

### DCs Cultured on the ZnO/Al_2_O_3_ Core–Shell Nanowire Platform

**Figure 2:**
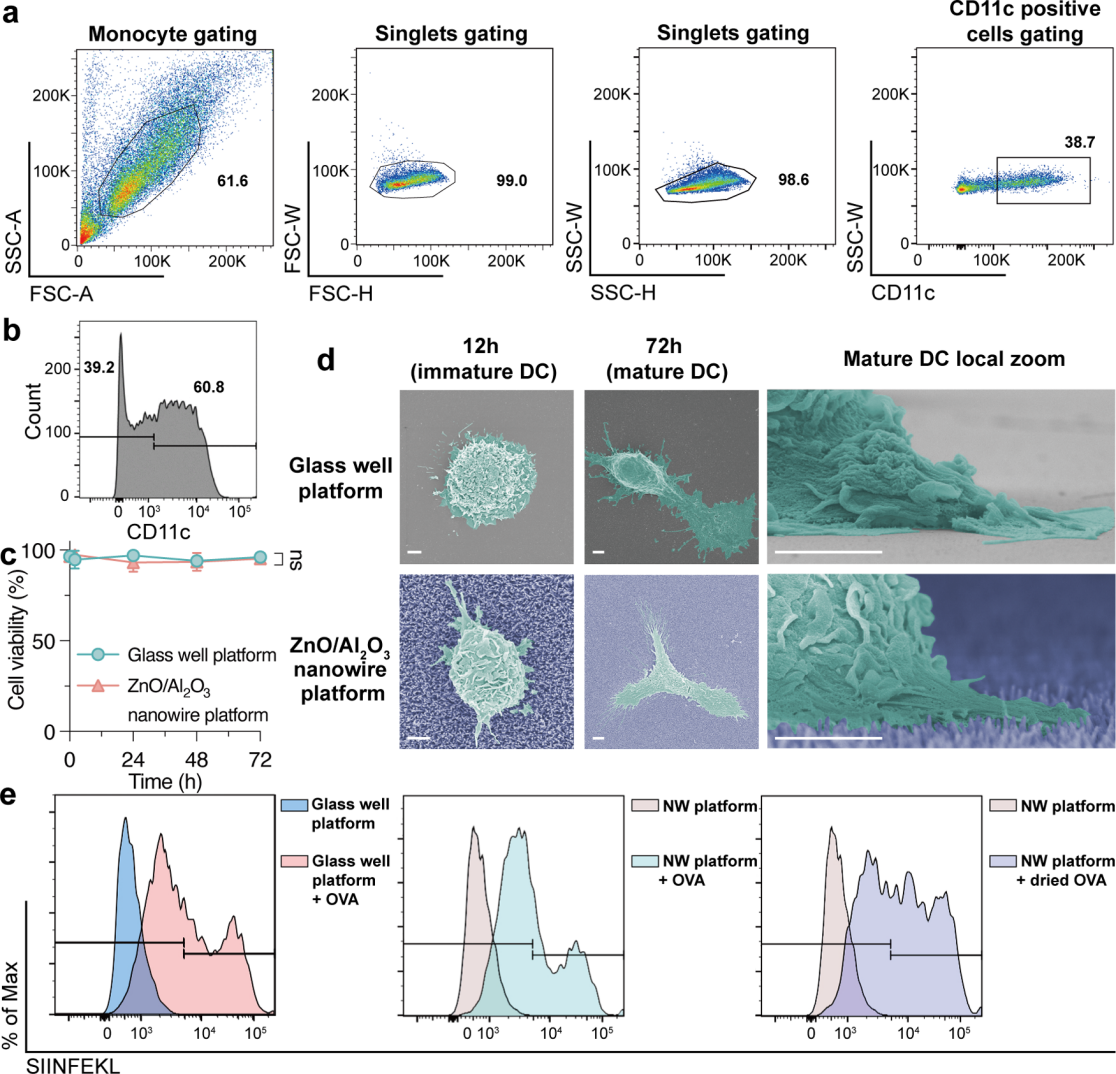
Culture and antigen presentation of DCs on the ZnO/Al_2_O_3_nanowire platform. (a) Gating scheme for identifying CD11c-positive subsets by flow cytometry. (b) Verification of differentiation from monocytes to immature DCs by flow cytometry. The histogram represents the CD11c-positive cells. (c) DC viability over time for the glass well platform and ZnO/Al_2_O_3_ nanowire platform. Error bars show the SD for an individual experiment (*N* = 3). The *p* value was calculated by an unpaired two-tailed *t*-test (ns, not significant). (d) Field emission scanning electron microscope (FESEM) images of DCs on glass and ZnO/Al_2_O_3_ nanowires; scale bars, 2 μm. (e) Cross-presentation of the SIINFEKL/H-2Kb complex of DCs was monitored by flow cytometry.

Having confirmed that nanowires were capable of EV capture and cell proliferation, we carried out cell differentiation and proliferation of DCs on the ZnO/Al_2_O_3_ core–shell nanowire platform. DCs are the most efficient APCs of immune cells, as they connect innate and adaptive immunity by critically regulating T-cell responses. Monocytes are an innate immune cell population that is able to differentiate into macrophages and DCs. We incubated monocytes collected from the bone marrow of mice and induced them to differentiate into immature and mature DCs. CD11c, which is a transmembrane protein and a widely used marker for DCs,^43^ was used to assess the differentiation level of DCs. A gating scheme was used to identify CD11-positive cells by flow cytometry (Figure 2a). The fluorescence of CD11c measured by flow cytometry confirmed that the differentiation of monocytes into immature DCs was induced, and about 61% of the cells expressed CD11c with high fluorescence (Figure 2b). We also showed that the DCs cultured on the platform maintained high viability, and there was no significant difference between cells cultured on the glass well platform and on the nanowire platform (Figure 2c).

To gain a deeper understanding of the culturing conditions of DCs on the nanowire platform, we made a microscopic characterization of the DCs using FESEM. From the FESEM images, the immature DCs (without lipopolysaccharide (LPS)) survived in a spherical shape on both the glass well platform and the nanowire platform, which was characteristic of immature DCs (Figure 2d). We also confirmed that mature DCs adhered to both the glass well platform and the nanowire platform and had a rough surface with multiple pseudopodia on each substrate (Figure 2d). It is known that for immature DCs, the circular shape is more likely to result in a greater number of particles being phagocytized, and that for immature DCs with a low level of activation and a high phagocytic capacity, they could take up antigens and mature, thereby acquiring a more active phenotype.^44,45^ Compared to immature DCs, mature DCs, which had longer dendrites, had less pronounced phagocytic ability; on the other hand, mature DCs are known to be more motile to interact with lymphocytes, awaken T lymphocytes, trigger a strong immune response, and destroy tumors.^46,47^ Considering the state of the DCs indicated by the FESEM observations, we assumed that EVs captured on the surface of the nanowire platform had a chance to contact DCs directly. DCs have been shown to use two distinct mechanisms for antigen capture: macropinocytosis and the mannose receptor,^48^ one of which, macropinocytosis, is constitutive and allows continuous internalization of large amounts of fluid. Therefore, we thought that DCs could absorb not only the surface-attached EVs on the nanowire tip of the nanowire platform but also the EVs caught on the nanowire side of the nanowire platform.

### Antigen Presentation of DCs on the ZnO/Al_2_O_3_ Core–Shell Nanowire Platform

DC immunotherapy involves loading DCs with peptides, DNA, RNA, or patient tumor cells to present tumor-associated antigens.^49^ Then, we investigated whether the peptides on nanowires were presented to DCs. Ovalbumin peptides, which are generally synthesized in the same manner as the presentation of the MHC class I H-2Kb allele, are known to be presented by DCs. The ovalbumin peptide was added to the nanowire platform, and the platform was dried in a nitrogen gas flow so that the nanowires were coated with the ovalbumin peptide. Immature DCs were seeded on the nanowire platform, and LPS was added 12 h later to induce the formation of mature DCs. Finally, the matured DCs were contacted with ovalbumin peptide for 60 h and harvested on the nanowire platform by pipetting. Flow cytometry was used to assess the presence of SIINFEKL (OVA 257–264) by H-2Kb monoclonal antibodies, and it showed that among CD11c-positive subsets, DCs presented antigen with ovalbumin peptide on the glass well platform and the nanowire platform (Figure 2e). The nanowire platform had no effect on the antigen presentation process compared to the process on the glass well platform, similar to a normal cell culture dish; immature DCs took up LPS suspended in the solution to mature DCs, and then the matured DCs took up peptide coated on the nanowire platform, decomposed it inside the cell, and presented the antigen on H-2Kb. The ZnO/Al_2_O_3_ core–shell nanowires provided a significant platform for DC differentiation, proliferation, antigen uptake, and antigen presentation.

### EV Capture on the ZnO/Al_2_O_3_ Core–Shell Nanowire Platform

**Figure 3:**
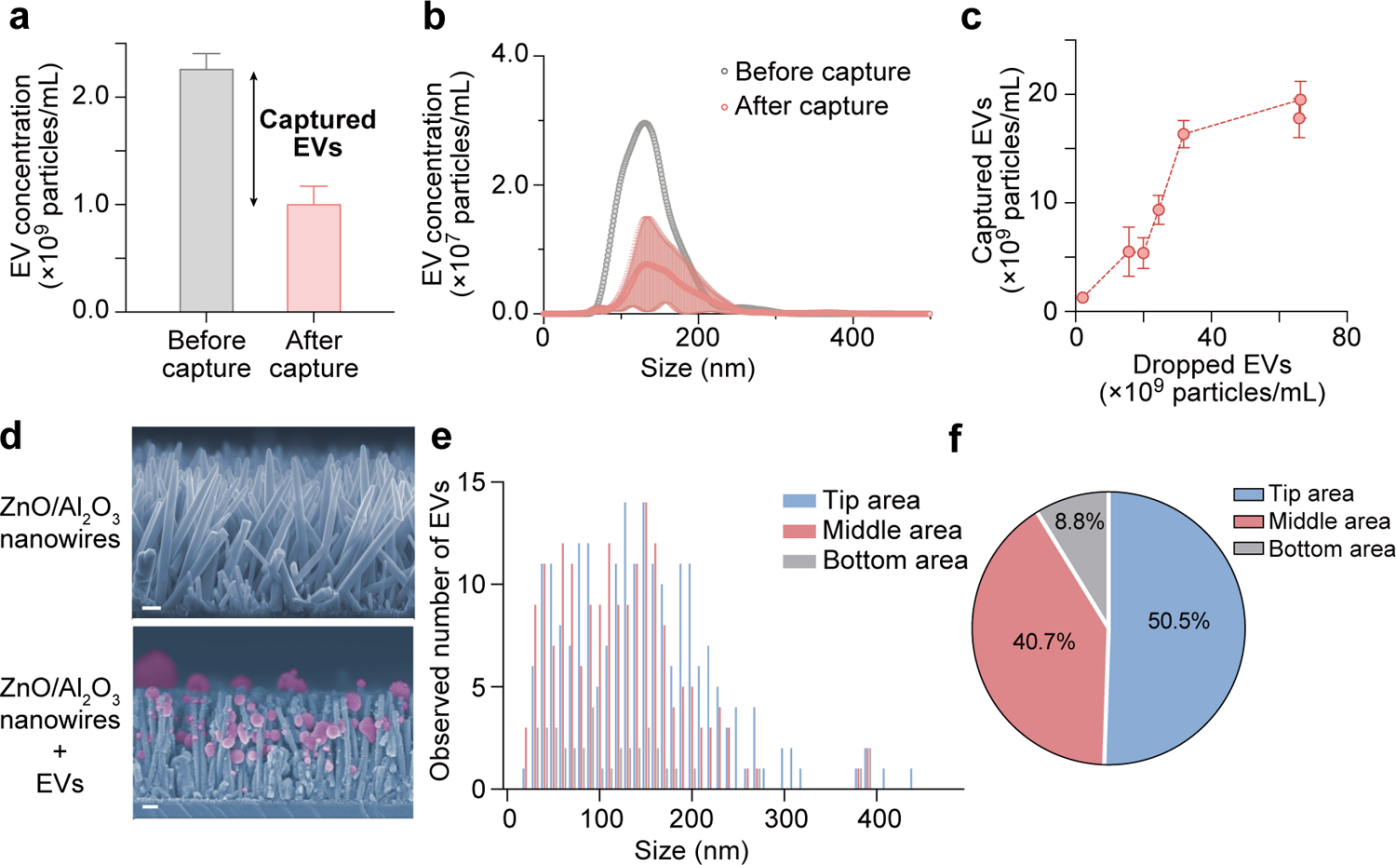
EV capture on the ZnO/Al_2_O_3_ nanowire platform. (a) EV capture efficiency of ZnO/Al_2_O_3_ nanowires. Error bars show the SD for an individual experiment (*N* = 3). (b) Size distribution of EVs before and after incubation on the nanowire platform. Error bars show the SD for an individual experiment (*N* = 4). (c) Maximum number of EVs that the ZnO/Al_2_O_3_ nanowire platform caught. Error bars show the SD for an individual experiment (*N* = 3). (d) FESEM images of the ZnO/Al_2_O_3_ nanowire platform-captured EVs; scale bars, 200 nm. The nanowires and EVs were highlighted in blue and pink, respectively. (e) Number of captured EVs (*N* > 400) observed from FESEM images on different parts of the nanowires and EV size distribution. (f) Ratio of EVs captured on different parts of the nanowires analyzed from (e).

In addition to detecting the membrane protein CD63 on EVs (Figure 1b), EV capture was investigated by using nanoparticle tracking analysis (NTA). The collected GL261 cell supernatant was measured by NTA, and then the supernatant was dropped onto the nanowire platform, and the supernatant after 20 h was measured again with NTA. When the introduced EV concentration was approximately 2.0 × 10^9^ particles/mL, approximately 60% of the EVs were collected (Figure 3a). The size distribution of EVs before and after incubation on the nanowires demonstrated that the size range of captured EVs was 30–200 nm, implying that the nanowires captured small EVs (sEVs)^50^ (Figure 3b). The detection of CD63, a type of sEV marker, and the capture of EVs ranging from 30 to 200 nm provide evidence that this nanowire platform is effective in capturing CD63+ sEVs. Moreover, we found that the number of captured sEVs increased as the input particle concentration increased, and the nanowire platform could capture at most about 1.8 × 10^10^ particles/mL (Figure 3c). Based on the area of 186 mm^2^ of this platform and the area of 19.6 μm^2^ for a single cell, we estimated that approximately 1.9 × 10^3^ EVs could contact per single cell.

The SEM images confirmed that sEVs were adsorbed on the nanowires onto which the cell supernatant was dropped (Figure 3d). These sEVs retained their spherical shape, and they were present on various positions of the nanowires. Small EVs have a diameter of 30–200 nm, and therefore, the spacing between nanowires in the network is the key point for sEV capture. Small EVs could be captured at different locations on the nanowires; for example, being captured when entering the space between the nanowires, being captured when adsorbed on the nanowire sides, and being captured when riding on the nanowire tips. In addition, we divided the length of the nanowires into three equal parts and evaluated the number of EVs captured at each position (Figure 3e). The tip area of the nanowires captured approximately 50% of the sEVs, while the bottom area captured only about 10% (Figure 3f). Since DCs were cultured on the top of the nanowire platform, and the majority of sEVs were captured on the upper part of the nanowires near DCs, we have no doubts that the nanowire platform provides a highly efficient way for sEVs to make contact with DCs. Based on this, sEVs can be further phagocytized to present antigens.

### Coculture of Captured EVs with DCs and Uptake of EVs into DCs

**Figure 4:**
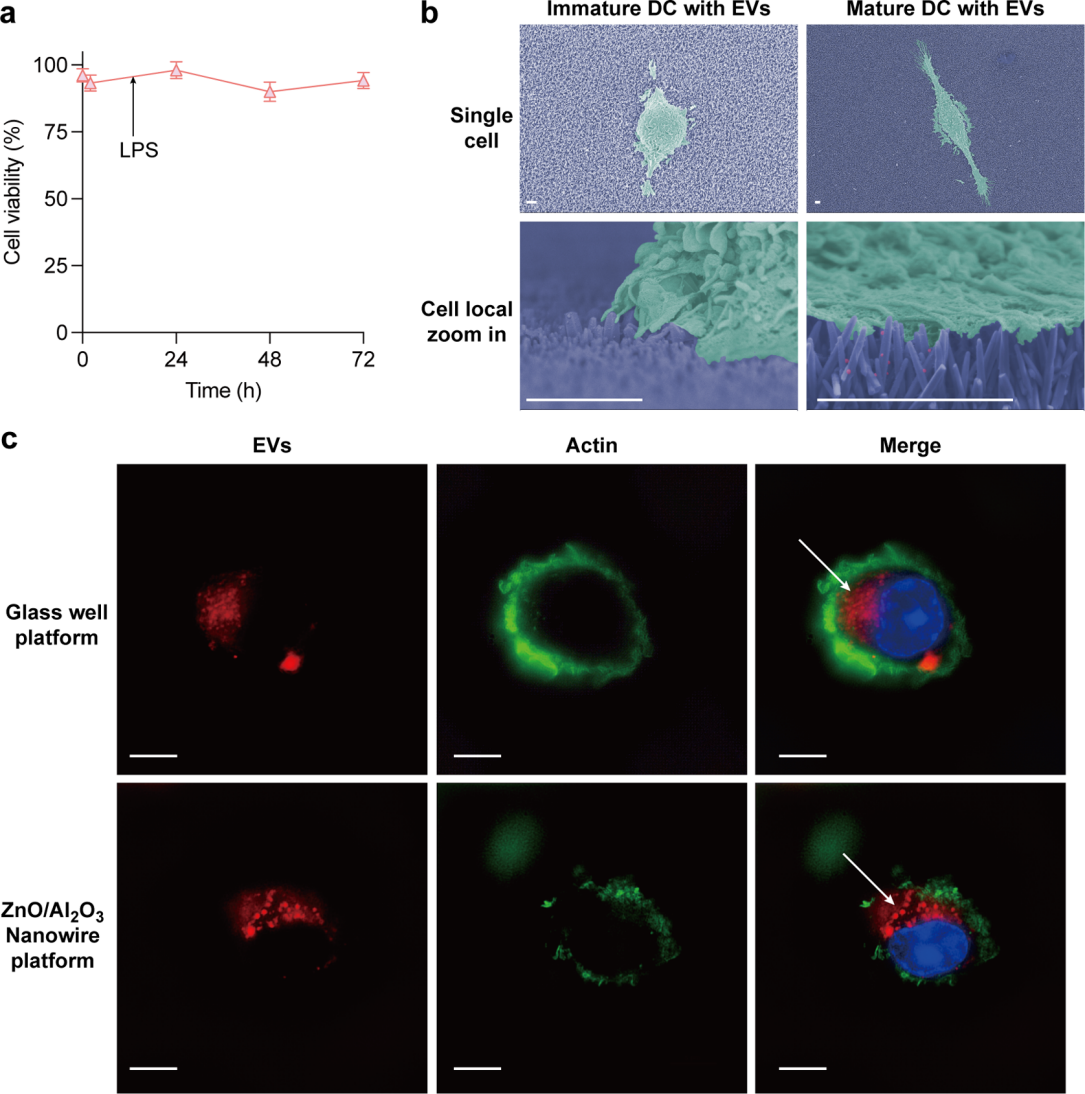
Incorporation of GL261 cell-derived EVs in DCs. (a) DC viability on nanowires with GL261 cell-derived EVs. The arrow indicates the addition of LPS at 12 h. Error bars show the SD for an individual experiment (*N* = 3). (b) FESEM images of DCs on nanowires; scale bars, 2 μm. (c) Uptake of EVs of DCs; scale bars, 5 μm. EVs were visualized with DiR antibody (red), actin was visualized with Alexa Fluor 588 phalloidin (green), and cell nuclei were stained with Hoechst 33342 (blue). The white arrows point to the incorporated GL261 cell-derived EVs in DCs.

We examined whether the presence of EVs captured by the ZnO/Al_2_O_3_ core–shell nanowires affected the DC viability and maturation. Tumor-derived EVs have been reported to induce apoptosis of cells,^51,52^ but the viability of DCs did not decrease in our study; the presence of EVs did not affect cell growth (Figure 4a). It is most likely that the differentiation was induced for the monocytes to be immature DCs, and the immature DCs took up LPS suspended in the solution to mature DCs. SEM results also confirmed that the cells changed from a spherical shape to mature cells 60 h after LPS addition (Figure 4b). From these results, EVs captured by the nanowire platform did not affect the function of the DCs, leading us to think that the nanowire platform could also be a tool for the differentiation and proliferation of immune cells.

Finally, we investigated whether the sEVs captured by the nanowire platform were incorporated into the DCs. Since DCs take up the antigen into the cell mainly by macropinocytosis, a type of endocytosis, and the antigen is decomposed and presented on the cell membrane MHC, DCs have to take up the sEVs into the cells to present the antigen from the sEVs. It has been reported that DCs generally have prominent antigen uptake at the immature stage and that the uptake is most active 30–45 min after LPS addition.^53^ In our research, 1 h after the addition of LPS to immature DCs on the sEV-captured nanowire platform, DCs were pipetted off the nanowire platform for super-resolution observation. As a control experiment, EVs were collected by ultracentrifugation, stained, and added to the medium for DCs cultured on the glass well platform at the same particle concentration as on the nanowire platform. The results from the merged images confirmed that EVs were taken up into the cells in both groups (Figure 4c), indicating that DCs took up EVs on this ZnO/Al_2_O_3_ nanowire platform. From the shape of the DCs, which are round without dendrites, we assumed that the cells were still close to immaturity as they were immobilized only 1 h after LPS addition and therefore not sufficiently mature, and this is consistent with the fact that DCs have the capacity for antigen uptake at an immature stage. Overall, our findings suggest that the nanowire platform is an effective tool for capturing and studying EVs and that DCs can incorporate EVs captured on this platform.

We further investigated whether EVs from ovalbumin-expressing GL261 (GL261-OVA) cells directly induced naive CD8 T cells to become cytotoxic T cells. We conducted the induction of cytotoxic T cells by culturing CD8+ T cells on a nanowire platform that collects GL261-OVA-derived EVs. Direct administration of EVs failed to elicit cytokine release from T cells, including IL-2 and IFN-γ (Figure S3). This outcome is likely due to the absence of the costimulatory factor CD86 on CD8 T cells. Therefore, other APCs that express CD86, such as macrophages, may be more suitable for this purpose. Since DCs are known to be potent APCs, our platform is poised to function as an artificial lymph node where DCs can mature and efficiently present tumor-specific antigens.

## Conclusions

In this research, we have demonstrated the realization of a platform for EV capture and the simultaneous differentiation and proliferation of DCs. Conventional ZnO nanowires effectively captured the EVs, but they were proved to be cytotoxic in this study, which is consistent with the literature;^54−56^ however, it is possible to remove the toxicity by forming a protective film using another metal oxide, which will satisfy EV capture and cell culture requirements simultaneously. DCs are generally considered the starting point of acquired immunity, and in conventional DC vaccine therapy, tumor tissue is administered to DCs to take up and present the tumor-associated antigen. When these DCs are reintroduced into the patient’s body, they present antigens to CD8^+^ T cells to induce antigen-specific cytotoxic T cells to eliminate specific cancer cells. Since EVs from cancer patients can be easily isolated from body fluids using nanowires and DCs can be isolated from blood and induced to mature in vitro, immunotherapy using EVs and DCs will be able to achieve the goal of noninvasive treatment. Furthermore, our nanowire platform allows for continued cell culture, suggesting that studying the effect of EVs on cell differentiation and proliferation can be achieved more easily compared to conventional methods. Our ZnO/Al_2_O_3_ core–shell nanowire platform enabled the collection of 60% or more of the EVs in cell supernatant, while DCs cultured on the nanowire platform maintained the cell shape and carried out the cell function without interference, and these DCs could be induced to become mature cells and present antigen along with antigen uptake. We used a single platform to capture EVs and produce specific antigen-presenting DCs with high efficiency and noninvasiveness. Although we still need to conduct further experiments to realize immunotherapy on the nanowire platform, we anticipate that our platform will open up new possibilities for noninvasive and personalized EV-based diagnosis and immunotherapy, which have been inaccessible until now.

## Materials and Methods

### Fabrication of the Nanowire Platform

To culture cells on a nanowire platform, we used a 24-well plate as the culturing tool. A glass substrate (Fujifilm Wako Chemicals, Tokyo, Japan) the same size as the 24-well plate (Corning Life Sciences, NY, USA) was sterilized with sulfuric acid (Fujifilm Wako Chemicals) and hydrogen peroxide solution (Fujifilm Wako Chemicals) at a ratio of 3:1 for 30 min and then rinsed with ultrapure water. A thin-film layer of ZnO was deposited on the sterilized glass substrate by radio frequency sputtering (SVC-700RF I, Sanyu Electron Co., Ltd., Tokyo, Japan) as a seed layer for nanowire growth. The ZnO nanowires were grown using the hydrothermal synthesis method^29^ with 30 mM hexamethylenetetramine (HMTA; Wako Pure Chemical Industries, Ltd.) and 30 mM zinc nitrate hexahydrate (Alfa Aesar, MA, USA) at 95 °C for 3 h. Then, the substrate was rinsed with ultrapure water and dried in a nitrogen gas flow. After fabrication of the ZnO nanowires, an ALD system (Savanna G2, Ultratech Inc., CA, USA) was used to deposit a thin layer of metal oxides to fabricate the core–shell nanowires, as described elsewhere.^27,29^ Finally, the glass substrate on which the nanowires were grown and got an oxide layer by ALD was sterilized using the CoolCLAVE Laboratory Bench Top Sterilizer (Genlantis Inc., CA, USA) and adhered to a 24-well plate using a Kwik-Sil (World Precision Instruments, FL, USA) to make the core–shell nanowire platform for EV capture and cell culture. Note that the glass substrate on which the ZnO nanowires were grown that adhered to a 24-well plate was the ZnO nanowire platform, and only a glass substrate adhered to a 24-well plate was the glass well platform.

### Collection of EVs from GL261 Cells

The GL261 cells used were provided by Professor Atsushi Natsume of Nagoya University. The cells were cultured in an incubator in DMEM (Thermo Fisher Scientific Inc., MA, USA) with 10% fetal bovine serum (Thermo Fisher Scientific Inc.) and 1% penicillin/streptomycin (Thermo Fisher Scientific Inc.) at 37 °C, in 5% CO_2_. When the cells reached about 80% confluence, we changed to Advanced DMEM (Thermo Fisher Scientific Inc.) to continue culturing for another 4 days. Finally, the cell supernatant was collected and centrifuged at 300*g* at 4 °C for 10 min and 2000*g* at 4 °C for 10 min to remove dead cells and cell debris. After filtering through a 0.22 μm filter (Merck, Darmstadt, Germany), we obtained the supernatant containing the EVs.

### Detection of Captured EVs on the Nanowire Platform by a Plate Reader

A 1 mL aliquot of the cell supernatant obtained from the cultured GL261 cells was dropped onto the core–shell nanowire platform sterilized with the CoolCLAVE. After incubating at 37 °C, in 5% CO_2_ for 20 h, the supernatant solution was gently removed by pipetting, and the EVs were captured by the nanowires. The captured EVs were washed with 200 μL of PBS (Thermo Fisher Scientific Inc.) and blocked with 10% Blocking One (Nacalai Tesque Co., Ltd., Kyoto, Japan) and 0.5% Tween 20 (PanReac AppliChem, IL, USA) in PBS for 10 min. After washing 3 times with PBS, antimouse CD63 antibody (Biolegend, CA, USA) was incubated with the EVs for 20 min at a concentration of 2/100 μL in PBS per well. Following this procedure, the antibody was removed and washed with PBS again. Finally, 100 μL of PBS was added to prevent drying during the measurement. The fluorescence intensity was detected with a plate reader (Tecan Group Ltd., Männedorf, Switzerland) with an excitation wavelength of 614 nm and an emission wavelength of 664 nm. The parameters were set as follows: gain, 140; number of flashes, 30; integration time, 40 μs; Z-position mode, manual; multiple reads per well (circle (filled)), 4 × 4; and border, 1000 μm.

### NTA Measurements

The number of EVs was measured and quantified by tracking the Brownian motion of single particles using a NanoSight LM10 (Malvern Panalytical, Worcestershire, UK) equipped with a sample chamber, a 405 nm laser, and a high-sensitivity scientific complementary metal-oxide-semiconductor camera. Each sample was injected into the chamber using a sterile syringe (Terumo, Tokyo, Japan) until the liquid reached the tip of the nozzle. Live monitoring of NTA acquisition was performed using a syringe pump loading system (Isis Co., Ltd., Osaka, Japan) at a rate of 5 μL/min. All measurements were performed at room temperature. The samples were diluted empirically with advanced DMEM to achieve 30–100 particles/frame. Data were collected from 3 × 60 s videos recorded with a viscosity value of 0.94 cP (advanced DMEM), camera level of 15, detection threshold of 5, and all other parameters set as the default. Data were processed with NanoSight NTA 3.2 software.

### Cell Viability Assay

The GL261 cells were pipetted from the platforms and diluted to the appropriate concentration. Propidium iodide (Life Technologies, MA, USA) and Calcein AM (Life Technologies) were used to separate the live and dead cells. Cell count was performed with an automatic cell counter (Thermo Fisher Scientific Inc.). Cell viability was evaluated by the ratio of live cells to the total cells.

### DC Culture

Monocytes collected from the bone marrow of C56BL/6 mice (6–10 week-old females; CLEA Japan, Tokyo, Japan) were cultured in RPMI 1640 medium supplemented with 10% fetal bovine serum and 20 ng/mL GM-CSF (PeproTech, Cranbury, NJ, USA) in a humidified incubator at 37 °C, in 5% CO_2_ for 7 days to induce differentiation into immature DCs. The medium was changed 2–3 times a week, 2.5 × 10^5^ immature DCs were dropped onto the platforms, and 1 μg/mL LPS (Sigma-Aldrich, MO, USA) was added for the induction of mature DCs.

### SIINFEKL Assay of DCs

DCs were collected and centrifuged at 300*g* for 3 min at 4 °C. After washing the cells with PBS, TruStain FcX PLUS (Biolegend, CA, USA) was added at 1:100 dilution, and the cells were incubated at 4 °C in the dark for 10 min. After the cells were washed with washing buffer and centrifuged at 300*g* for 3 min at 4 °C, SIINFEKL antibody (Biolegend) and diluted CD11c antibody (Biolegend) were added at 1:100 dilution each, and the mixture was incubated at 4 °C in the dark for 15 min. After that, the cells were evaluated using an LSRFortessa X-20 and FlowJo software (BD Biosciences, CA, USA). Monocytes were isolated by gating on a forward scatter (FSC)–area/side scatter (SSC)-area plot, followed by exclusion of any clumps of multiple cells on FSC-hight/FSC-width and SSC-hight/SSC-width plot. From isolated singlet monocyte, the CD11c-positive subset was selected as DC.

### FESEM Measurements

Immature DCs were cultured on the nanowire platform and the glass well platform, and LPS was added to induce the mature DCs. Thereafter, the cells were immobilized and observed. Each platform was washed with PBS and prefixed for 2 h with 1% paraformaldehyde phosphate buffer (Nacalai Tesque Co., Ltd.) diluted with PBS. For postfixation, osmium tetroxide (Tokyo Chemical Industry Co., Ltd.) was diluted with PBS to 0.5% and incubated with each platform for 15 min. After the fixed platforms were washed with PBS, it was soaked in 50, 70, 80, 90, 95, and 100% ethanol solution for 15 min each, and soaked in 100% ethanol/amyl acetate = 1:1 solution for 15 min. Finally, the platforms were immersed in amyl acetate solution (Tokyo Chemical Industry Co., Ltd.) for 15 min and dried with a critical point dryer JCPD-5 (JEOL, Tokyo, Japan) for 2 h. Platinum sputtering was done for 15 s, and then, the FESEM observations were made using a Supra 40VP (Carl Zeiss, Jena, Germany) operated at an acceleration voltage of 5 kV. The FESEM images of EVs were obtained by the same method.

### DC Culture on Nanowires which Captured EVs

A 1 mL per well aliquot of GL261 cell supernatant containing EVs was dropped onto the ZnO/Al_2_O_3_ nanowire platform and incubated for 20 h to capture the EVs. After that, the supernatant solution was gently removed by pipetting, the residue was washed with PBS, a suspension of DCs in RPMI 1640 medium was added dropwise to the amount of 1 mL per well, and the cells were cultured on the EVs.

### Uptake of EVs by DCs (Super-Resolution Microscopy)

A 200 μL aliquot of the GL261 cell supernatant was dropped onto the ZnO/Al_2_O_3_ nanowire platform, and it was incubated for 20 h. The supernatant was removed gently by pipetting, the nanowire platform was washed with PBS, and then it was blocked with 200 μL of blocking agent (PBS/BSA/tween 20 = 100:10:0.5) for 20 min. After washing with PBS, the DiR (Diluent/DiR = 1000:2) (Dojindo Molecular Technologies, Inc., Kumamoto, Japan) was added for incubation with the EVs for 20 min and then washing with PBS again. Subsequently, 1 × 10^5^ immature DCs were dropped onto the nanowire platform. After culturing for 24 h, 1 μg/mL LPS was added dropwise to induce maturity in the immature DCs. After 1 h, the mature DCs were collected from the nanowire platform and cultured on a glass-based dish overnight in the incubator. After the cells adhered firmly, the supernatant was removed. After the cells were washed with PBS, 4% PFA (Nacalai Tesque Co., Ltd.) was used to immobilize the cells by incubating them at 4 °C for 30 min. Hoechst 33342 (Dojindo Molecular Technologies, Inc.) and Alexa Fluor 588 phalloidin (Thermo Fisher Scientific Inc.) were diluted with PBS to stain the cell nuclei and actin at 4 °C for 1 h. Afterward, the cells were washed with PBS, and they were observed with a super-resolution microscope.

As a control experiment, the GL261 cell supernatant containing EVs was collected and precipitated by ultracentrifugation at 110,000*g* for 80 min at 4 °C with the CS150FNX ultracentrifuge (Hitachi Co., Ltd., Tokyo, Japan). Collected EVs were washed with Advanced DMEM and ultracentrifuged at 110,000*g* for 80 min at 4 °C again. Then, the concentration was adjusted to the same concentration as that captured by the nanowire platform and stained by DiR, as described before. Immature DCs were cultured on a glass-bottom dish overnight, and that was followed by dropping the stained EVs onto the cells.

### T Cell Culture on Nanowires, which Captured EVs

OT-1 mice are genetically engineered to possess a specific receptor for the SIINFEKL peptide of ovalbumin, leading to IFN-γ production upon H-2Kb-restricted recognition of the SIINFEKL presentation. Their splenic CD8^+^ T cells were cultured with CD3/CD28 beads for 3 days. The cultured CD8^+^ T cells (2.5 × 10^5^ cells) were then adhered to the nanowires capturing EVs from the supernatant of either wild-type GL261 (GL261-WT) or GL261-OVA cells. Golgi was stopped with Brefeldin A solution (1000× dilution) to prevent IFN-γ release. After 3 days of culture, cells were collected by pipetting, and IFN-γ production was evaluated by flow cytometry. Additionally, a drop of phorbol 12-myristate 13-acetate (PMA) at 50 ng/mL (2000× dilution) and 1 μM Lonomycin (1000× dilution) was used as a positive control.

## Supporting Information Available

Characterization of ZnO/Al_2_O_3_ (core/shell) nanowires and investigation of optimal aluminum oxide film thickness (PDF)
