# Supplementary material for: On-Site Stimulation of Dendritic Cells by Cancer-Derived Extracellular Vesicles on a Core–Shell Nanowire Platform
Source: ACS Appl Mater Interfaces. 2024 May 28;16(23):29570–80. doi: 10.1021/acsami.4c00283 (PMC11181270; doi:10.1021/acsami.4c00283)
Supplement: Supplementary file 1 — am4c00283_si_001.pdf [file am4c00283_si_001.pdf]

Supporting Information

On-site stimulation of dendritic cells by cancer-derived extracellular vesicles on a core-shell nanowire platform

Min Zhang[1], Miki Ono[1], Shota Kawaguchi[1], Mikiko Iida[1], Kunanon Chattrairat[2], Zetao Zhu[2], Kazuki Nagashima[3], Takeshi Yanagida[4], Junya Yamaguchi[5], Hiroyoshi Nishikawa[5,6], Atsushi Natsume[7,8], Yoshinobu Baba[1,7,9*], Takao Yasui[1,2,7,9*]*

[1]Department of Biomolecular Engineering, Graduate School of Engineering, Nagoya University, Furo-cho, Chikusa-ku, Nagoya 464-8603, Japan.
[2]Department of Life Science and Technology, Tokyo Institute of Technology, Nagatsuta 4259, Midori-ku, Yokohama 226-8501, Japan.
[3]Research Institute for Electronic Science (RIES), Hokkaido University, Kita, Sapporo, Hokkaido 001-0020, Japan.
[4]Department of Applied Chemistry, Graduate School of Engineering, The University of Tokyo, 7-3-1 Hongo, Bunkyo-ku, Tokyo 113-8656, Japan.
[5]Department of Immunology, Nagoya University Graduate School of Medicine, Nagoya 466-8550, Japan.
[6]Division of Cancer Immunology, Exploratory Oncology Research and Clinical Trial Center (EPOC), National Cancer Center, Chiba 277-8577, Japan
[7]Institute of Nano-Life-Systems, Institutes of Innovation for Future Society, Nagoya University, Furo-cho, Chikusa-ku, Nagoya 464-8603, Japan.
[8]Kawamura Medical Society, Gifu 501-3144, Japan
[9]Institute for Quantum Life Science, National Institutes for Quantum Science and Technology (QST), Anagawa 4-9-1, Inage-ku, Chiba 263-8555, Japan.

*Corresponding authors: (M. Zhang) E-mail: min.zhang@chembio.nagoya-u.ac.jp; (Y. Baba) E-mail: babaymtt@chembio.nagoya-u.ac.jp; (T. Yasui) E-mail: yasuit@bio.titech.ac.jp

The Supporting Information includes:

Figure S1. Characterization of ZnO/Al_2O_3 core-shell nanowires.
Figure S2. Investigation of optimal aluminum oxide film thickness.
Figure S3. Evaluation of CD8+ T cell activation by IFN-γ.

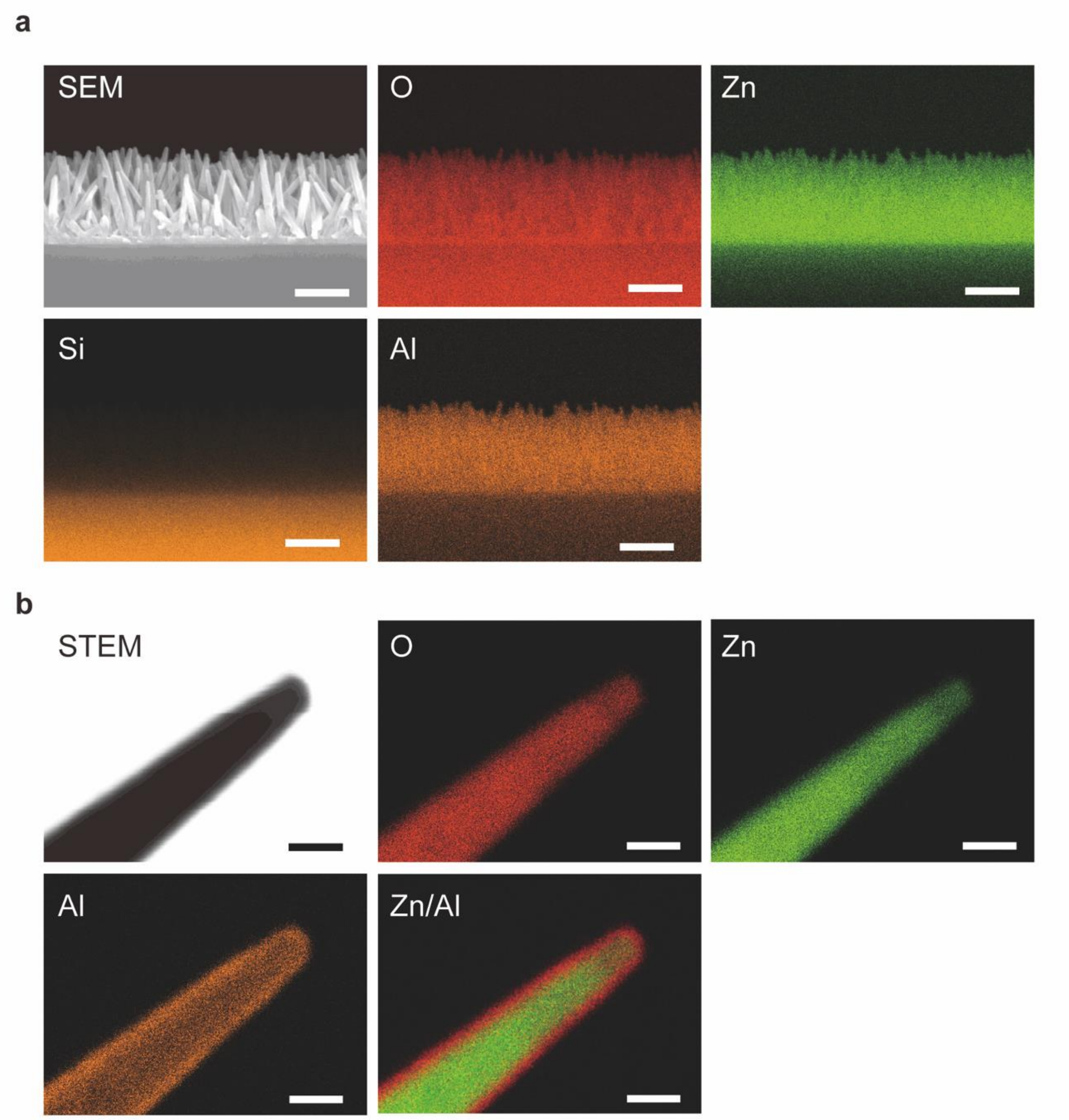

Figure S1. Characterization of ZnO/Al_2O_3 core-shell nanowires. (a) A cross-sectional FESEM image and corresponding EDS elemental mappings of the nanowires on quartz substrate; scale bar, 1 μm. (b) A STEM image of a nanowire and corresponding EDS elemental mappings; scale bar 100 nm. In (a) and (b), Zn, O, Si and Al are respectively highlighted as green, red, and orange for single elemental mappings. In (b), Zn and Al are respectively highlighted as green and red for dual elemental mappings.

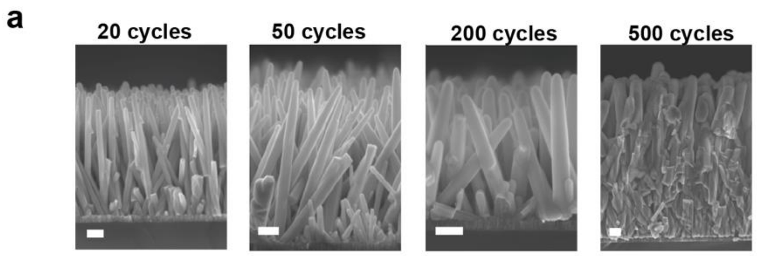

b

	20 cycles	50 cycles	200 cycles	500 cycles
Thickness	2 nm	5 nm	20 nm	50 nm
Length	1207 nm	1450 nm	1962 nm	2923 nm
Width	92 nm	96 nm	131 nm	231 nm
Interval	401 nm	301nm	176 nm	173 nm

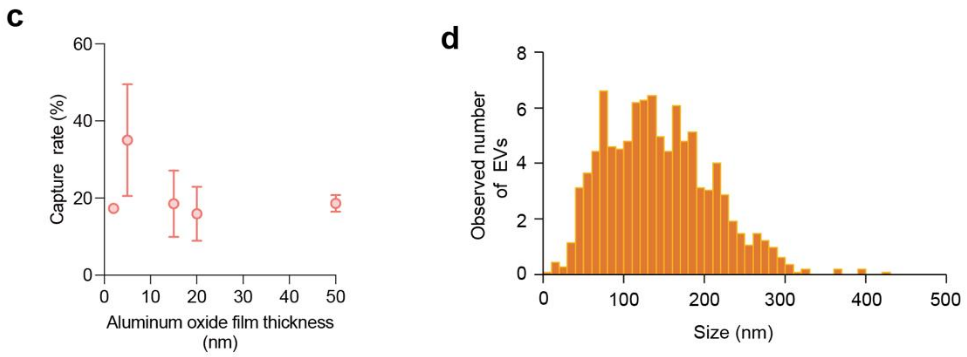

Figure S2. Investigation of optimal aluminum oxide film thickness. (a) FESEM images of nanowires with different numbers of aluminum oxide ALD cycles; scale bars, 200 nm. (b) The parameters of the nanowires with different numbers of aluminum oxide ALD cycles. (c) The capture rate for different numbers of ALD cycles which reflect thicknesses of the nanowires. Error bars show the SD for an individual experiment (N = 3). (d) Captured number of EVs observed from FESEM images and the EV size distribution.

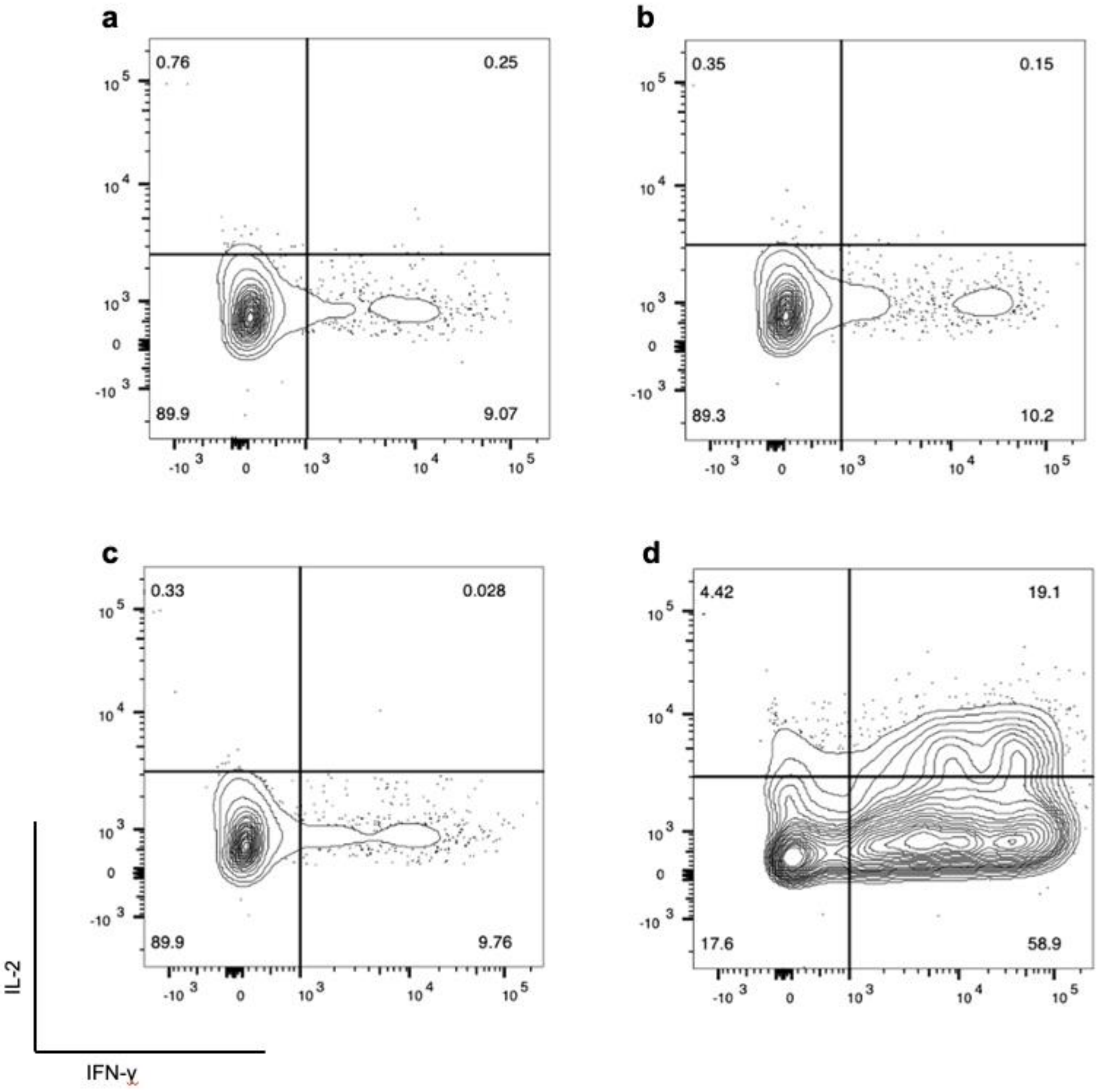

Figure S3. Evaluation of CD8+ T cell activation by IFN-γ. (a) EVs from GL261-OVA on the nanowire platform. (b) EVs from GL261-WT on the nanowire platform. (c) EVs from GL261-OVA using ultracentrifugation. (d) PMA+lonomycine
